# Economic evaluation of newly approved cetuximab biosimilars for first-line treatment of RAS/BRAF wild-type metastatic colorectal cancer in China

**DOI:** 10.3389/fonc.2026.1901037

**Published:** 2026-07-28

**Authors:** Rui Feng, Liman Huo, Ping Liang, Dong Li, Rong Cui, Ying Zheng, Qi Lv

**Affiliations:** 1 School of Disaster and Emergency Medicine, Tianjin University, Tianjin, China; 2 Department of Pharmacy, The Fourth Hospital of Hebei Medical University, Shijiazhuang, China

**Keywords:** biosimilars, cetuximab, cost-effectiveness, Markov model, metastatic colorectal cancer

## Abstract

**Background:**

Domestic cetuximab biosimilars have recently expanded the EGFR-targeted treatment landscape for RAS/BRAF wild-type metastatic colorectal cancer (mCRC) in China, but their economic value remains uncertain.

**Methods:**

A three-state Markov model was developed from the Chinese healthcare system perspective to evaluate newly approved cetuximab biosimilars for first-line RAS/BRAF wild-type mCRC. Clinical inputs were derived from two phase III trials (NCT04835142 and NCT03206151), with long-term survival extrapolated from reconstructed Kaplan–Meier data. Costs, quality-adjusted life-years (QALYs), and incremental cost-effectiveness ratios (ICERs) were estimated over a 10-year horizon using 14-day cycles, 4.5% discounting, and willingness-to-pay (WTP) thresholds of USD 29,241.86 and USD 43,862.79/QALY. Deterministic and probabilistic sensitivity analyses were conducted, and an exploratory contextual cross-trial assessment was performed.

**Results:**

Reference cetuximab plus chemotherapy yielded 1.60 QALYs at a cost of USD 246,542.51, compared with 1.53 QALYs and USD 231,648.11 for A140 plus chemotherapy, resulting in an ICER of USD 193,033.41/QALY. CMAB009 plus FOLFIRI yielded 1.83 QALYs at a cost of USD 214,917.27, compared with 1.46 QALYs and USD 148,659.96 for FOLFIRI alone, resulting in an ICER of USD 179,684.65/QALY. Both ICERs exceeded the predefined WTP thresholds. Exploratory contextual analyses suggested lower modeled costs for CMAB009 than A140, but results were sensitive to survival assumptions and should not be interpreted as formal comparative evidence.

**Conclusion:**

Under current pricing conditions, the economic value of cetuximab biosimilars varied across trial-defined comparisons and was mainly driven by drug acquisition costs.

## 1 Introduction

Colorectal cancer (CRC) is one of the most prevalent and lethal malignancies in China. In 2022, colorectal cancer accounted for approximately 517,000 new cases and 240,000 deaths in China, representing a substantial and growing public health burden (1).CRC has become the second leading cause of cancer-related death in China, following lung cancer (2). Notably, 20%–25% of patients are diagnosed with distant metastases at presentation, and nearly half develop metastatic disease during the course of illness (3).However, only approximately 40% of cases are detected at an early stage, and the prognosis deteriorates significantly once the tumor has spread. As such, metastatic colorectal cancer (mCRC) constitutes a major clinical and public health challenge in China.

For patients with RAS/BRAF wild-type mCRC, anti-epidermal growth factor receptor (EGFR) monoclonal antibodies such as cetuximab represent a standard first-line treatment option. Cetuximab has demonstrated significant improvements in progression-free (PFS) and overall survival (OS), particularly among patients with left-sided tumors (4).Major guidelines from both the National Comprehensive Cancer Network (NCCN) and the Chinese Society of Clinical Oncology (CSCO) recommend cetuximab-based regimens for these patients (5, 6).Notably, CSCO emphasizes EGFR-targeted combinations to improve survival outcomes. These recommendations are supported by robust clinical evidence, including meta-analyses and pivotal trials such as TAILOR, which confirmed that cetuximab plus chemotherapy yields superior outcomes compared to chemotherapy alone or non-EGFR/anti-VEGF strategies (7, 8).

Despite the established efficacy of anti-EGFR monoclonal antibodies, their high cost imposes a heavy burden on patients and healthcare systems (9).Biosimilars, offering comparable efficacy and safety to originators (10), have emerged as a cost-saving alternative. In China, biosimilars have expanded rapidly, with 49 biosimilars covering 18 molecular entities approved by March 2024, including (35) monoclonal antibody products (11). Meanwhile, centralized procurement and reimbursement negotiations have substantially shaped drug pricing in China (12), with national volume-based procurement achieving an average price reduction of 53% across 294 drug formulations37 and reimbursement negotiation reducing the cost per defined daily dose of targeted anticancer medicines by 48.9% (37). Therefore, China-specific acquisition prices and reimbursement policies are essential for evaluating newly approved EGFR-targeted biosimilars.

Although the economic value of reference cetuximab in first-line mCRC has been assessed, results remain inconclusive, with some Chinese studies reporting ICERs above the WTP threshold despite QALY gains (9). Two domestic biosimilars, CMAB009 and A140, were approved in 2024 and 2025, respectively (13, 14), based on phase III trials demonstrating comparable efficacy and safety to the reference product.

However, evidence regarding the economic value of these newly approved biosimilars in the Chinese healthcare system remains limited. The recent approval of multiple domestic cetuximab biosimilars has reshaped the EGFR-targeted treatment landscape in China, creating a need to evaluate not only individual biosimilar-based strategies but also the broader economic implications of available treatment options. Given differences in trial designs and chemotherapy backbones, the available evidence does not support a formal comparative effectiveness analysis across products. Nevertheless, an exploratory synthesis of existing phase III evidence may provide useful contextual information for healthcare decision-making.

Therefore, this study conducted health economic evaluations of newly approved cetuximab biosimilars for first-line treatment of RAS/BRAF wild-type metastatic colorectal cancer using evidence derived from Chinese phase III clinical trials. In addition, an exploratory cross-trial assessment was conducted to provide contextual information regarding the potential economic differences between available biosimilar strategies. The findings may provide evidence to support pharmacoeconomic evaluation and healthcare decision-making for EGFR-targeted therapies in China.

## 2 Methods

### 2.1 Study design and model overview

A model-based cost–utility analysis was performed to evaluate the economic value of cetuximab biosimilars for adult patients with RAS/BRAF wild-type metastatic colorectal cancer (mCRC) receiving first-line treatment in China. The present study used published clinical trial evidence as model inputs and did not involve the conduct of NCT04835142 or NCT03206151, nor the recruitment of trial participants. Clinical efficacy and safety inputs for the A140 analysis were derived from NCT04835142, whereas those for the CMAB009 analysis were derived from the published phase III trial NCT03206151 (13, 14).

The analysis was conducted from the perspective of the Chinese healthcare system and included direct medical costs only. A three-state state-transition Markov model was developed to estimate long-term costs and health outcomes. The model included three mutually exclusive health states: PFS, progressed disease (PD), and death. At model entry, patients were assumed to be in the PFS state and could remain progression-free, progress to PD, or die during subsequent cycles.

The cycle length was set at 14 days to match the administration schedules of FOLFOX6- and FOLFIRI-based treatment regimens evaluated in the model. A 10-year time horizon was adopted to capture long-term disease progression, costs, and health outcomes in patients with mCRC. This time horizon was supported by model projections showing that, at 10 years, the residual survival probabilities were 0.9% in the A140 group and 0.03% in the reference cetuximab group, indicating that most expected survival outcomes were captured within the model. Costs and health outcomes were discounted at an annual rate of 4.5% according to the 2025 Chinese Pharmacoeconomic Evaluation Guidelines (17).

Model outcomes included total costs, quality-adjusted life-years (QALYs), incremental costs, incremental QALYs, and incremental cost-effectiveness ratios (ICERs). According to the official 2025 Statistical Communiqué on National Economic and Social Development, China’s per capita gross domestic product (GDP) was CNY 99,665 (21). Using an exchange rate of USD 1 = CNY 6.81, the per capita GDP was estimated at USD 14,620.93. Therefore, willingness-to-pay (WTP) thresholds of two and three times the per capita GDP were USD 29,241.86/QALY and USD 43,862.79/QALY, respectively.

The primary analysis compared A140 plus mFOLFOX6 with reference cetuximab plus mFOLFOX6. In addition, CMAB009 plus FOLFIRI was evaluated against FOLFIRI alone using data from its published phase III trial NCT03206151. An exploratory cross-trial assessment was further performed to provide contextual information on the potential economic implications of available cetuximab biosimilars in China. Given the absence of head-to-head evidence between A140 and CMAB009, this exploratory assessment was not interpreted as a formal indirect treatment comparison.

### 2.2 Study overview

Clinical efficacy and safety inputs for the primary analysis were obtained from the published multicenter, randomized, double-blind phase III trial NCT04835142, which enrolled 688 patients. Participants were randomly assigned (1:1) to receive A140 plus mFOLFOX6 (n = 341) or reference cetuximab plus mFOLFOX6 (n = 347) ^14.^

Eligible patients were aged 18–75 years, had histologically confirmed RAS wild-type mCRC, and no prior systemic chemotherapy for metastatic disease. If previously treated with adjuvant/neoadjuvant therapy, a minimum interval of 6 months (non-platinum) or 12 months (platinum-based) was required. Other criteria included ECOG PS 0–1, life expectancy ≥16 weeks, and ≥1 measurable lesion (RECIST v1.1). Key exclusions were BRAF V600E mutation, prior VEGF/EGFR-targeted therapy, recent local treatment or surgery, brain/leptomeningeal metastases, significant comorbidities (e.g., uncontrolled diabetes, cardiovascular disease), active infections, renal replacement therapy, viral hepatitis, HIV, and pregnancy or lactation.

Drug dose and cost estimations followed regimen-specific dosing rules, with body surface area derived from the corresponding clinical trial populations and body weight estimated from trial sex distribution and national health statistics (15).

In this study, patients were randomized to receive either A140 or reference cetuximab, both combined with mFOLFOX6. The anti-EGFR agents were administered with a loading dose of 400 mg/m² on Day 1, followed by a weekly maintenance dose of 250 mg/m² (IV).

The mFOLFOX6 regimen included oxaliplatin 85 mg/m² and calcium folinate (CF) 400 mg/m² on Day 1, followed by 5-fluorouracil (5-FU) 400 mg/m² as an IV bolus and 2400 mg/m² as a continuous infusion over 48 ± 4 hours. Treatment was repeated every 14 days until disease progression or treatment discontinuation.

Upon progression, second-line treatment was modeled according to the 2023 CSCO guidelines and typically comprised FOLFIRI plus bevacizumab: irinotecan 180 mg/m², CF 400 mg/m², 5-FU 400 mg/m² (bolus) plus 2400 mg/m² (infusion over 48 ± 4 h), and bevacizumab 5 mg/kg, all on Day 1 of each 14-day cycle (16).

### 2.3 Model construction

**Figure 1:**
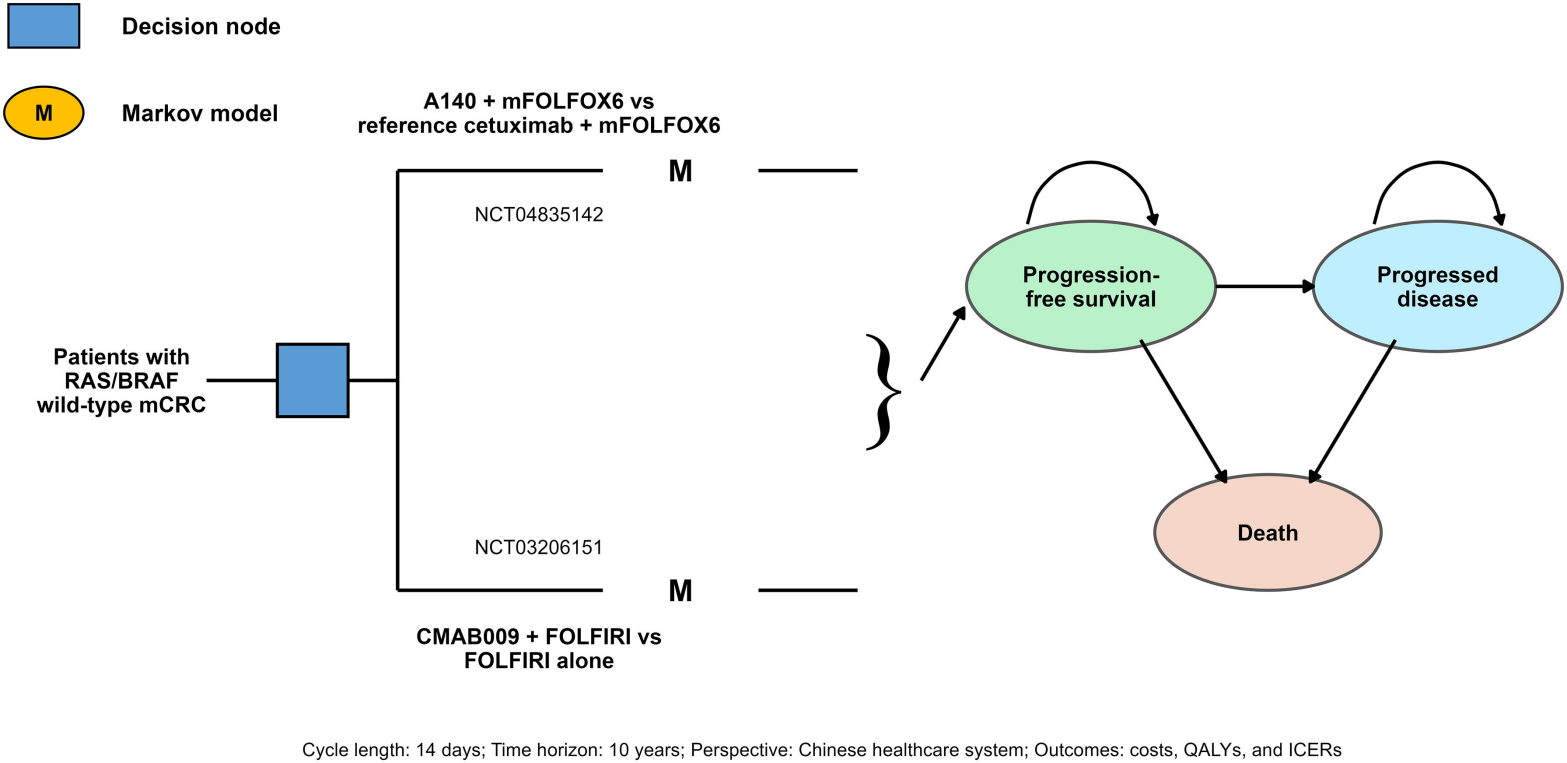
Structure of the economic model. The model included a decision node for first-line treatment strategies and a three-state Markov model consisting of progression-free survival, progressed disease, and death. The evaluated treatment strategies included A140 plus mFOLFOX6 versus reference cetuximab plus mFOLFOX6 and CMAB009 plus FOLFIRI versus FOLFIRI alone. Patients entered the Markov model in the progression-free survival state and could remain progression-free, progress to progressed disease, or die during subsequent 14-day cycles.

A three-state Markov model was developed in R software (version 4.4.2) using the “heemod” package to evaluate the cost-effectiveness of different treatment strategies for mCRC. The model consisted of three mutually exclusive health states: PFS, PD, and death, as shown in **Figure 1**.

Grade ≥3 adverse event (AE)-related costs and utility decrements were incorporated as one-time inputs in the first 14-day model cycle and weighted by the treatment-specific AE incidence rates reported in the corresponding clinical trials. The annual discount rate of 4.5% was applied at the cycle level according to the timing of cost and health outcome accrual. All costs and health outcomes, including AE-related costs and utility decrements, were discounted according to the cycle in which they accrued.

In addition, an exploratory contextual cross-trial assessment of CMAB009 and A140 was conducted using clinical data derived from two independent phase III trials involving Chinese patients with RAS/BRAF wild-type mCRC (13, 14). Scenario analyses were further performed to explore structural uncertainty associated with indirect cross-trial comparisons.

### 2.4 Effectiveness

For the primary A140 analysis, clinical efficacy data were obtained from the NCT04835142 phase III clinical trial. Long-term OS and PFS were extrapolated using parametric survival modeling approaches. Kaplan–Meier (KM) survival curves were digitized using WebPlotDigitizer (version 4.5), and individual patient data (IPD) were reconstructed using the “flexsurv” and “survHE” packages in R software (version 4.4.2).

Seven standard parametric survival models were evaluated, including exponential, Weibull, Gompertz, log-normal, log-logistic, gamma, and generalized gamma distributions. In addition, several flexible survival models were assessed, including fractional polynomial (FP), restricted cubic spline (RCS), Royston–Parmar (RP), generalized additive model (GAM), and mixture cure model (MCM) approaches. Both first-order (FP1) and second-order (FP2) FP models were explored, while RP models were evaluated under hazard, odds, and normal scales. Model selection was based on the Akaike Information Criterion (AIC), Bayesian Information Criterion (BIC), visual goodness-of-fit, and clinical plausibility (18, 19).

### 2.5 Costs and utilities

This study was conducted from the perspective of the Chinese healthcare system and included only direct medical costs. These included drug acquisition costs for cetuximab (originator and biosimilar agents), chemotherapy, disease management, best supportive care (BSC), terminal care, and adverse event (AE) management. Drug acquisition costs were obtained from the Wuxu Data Platform using median national bid-winning prices from January to June 2025. Median values were used to reduce the influence of regional price variation and better reflect nationally representative procurement prices in China (20). For cetuximab-based regimens, initial-cycle costs reflected the loading dose administered during the first 14-day cycle, whereas subsequent-cycle costs reflected maintenance dosing from the second cycle onward. Disease management costs included routine laboratory tests and imaging assessments. For parameters not directly available from this source, published literature was used as supplementary input (16, 22–30). All costs were converted to 2025 USD using an exchange rate of USD 1 = CNY 6.81.

Utility values for PFS and PD were derived from published cost-effectiveness studies and health technology assessment reports in mCRC (23–27, 29). Because health-state utility values were not directly collected in NCT04835142 or NCT03206151, published utility estimates from comparable mCRC populations were reviewed and summarized.

For the base-case analysis, utility inputs were selected according to clinical relevance to the modeled population, consistency with the PFS/PD health-state structure, and repeated use in previous mCRC economic evaluations. Priority was given to utility estimates from studies involving advanced or metastatic colorectal cancer, systemic treatment settings, anti-EGFR- or chemotherapy-based regimens, and HTA or model-based economic evaluations (23, 26, 27, 29). Across the reviewed studies, PFS utilities generally ranged from 0.75 to 0.85, while PD utilities ranged from 0.58 to 0.68. Based on these considerations, utility values of 0.78 for PFS and 0.60 for PD were applied in the base-case analysis. Detailed utility sources, clinical settings, reported values, and source characteristics are summarized in **Supplementary Table 3**.

The original utility elicitation instruments were not consistently reported across all source studies; therefore, the utility sources were summarized according to clinical setting and source type, such as literature-derived, HTA-derived, or model-based utility estimates. To simplify the model, only grade ≥3 AEs were included, and all events were assumed to occur during the first treatment cycle, consistent with commonly adopted pharmacoeconomic modeling approaches for acute treatment-related toxicities. AE incidence rates were obtained from the NCT04835142 clinical trial (14).

In the probabilistic sensitivity analysis (PSA), cost parameters were modeled using gamma distributions, whereas utility values and AE incidence probabilities were modeled using beta distributions to account for parameter uncertainty. For parameters without available variance estimates, ranges of ±25% from base-case values were assumed in sensitivity analyses. Detailed model inputs, parameter ranges, and distribution assumptions are summarized in **Table 1**.

**Table 1:** Base-case parameters, ranges, distributions, and sources used in the Markov model for A140 versus reference cetuximab.

Variable	Baseline value	Low	High	Distribution	Source
Costs of drugs ($/cycle)					
A140 initial-cycle cost	2148.1	1611.08	2685.13	gamma	Wuxu Data Platform,20; CSCO guideline,16
A140 subsequent-cycle cost	1652.38	1239.29	2065.48	gamma	Wuxu Data Platform,20; CSCO guideline,16
Cetuximab initial-cycle cost	1994.11	1495.58	2492.64	gamma	Wuxu Data Platform,20; CSCO guideline,16
Cetuximab subsequent-cycle cost	1533.93	1150.45	1917.41	gamma	Wuxu Data Platform,20; CSCO guideline,16
m-FOLFOX6	80.06	60.05	100.08	gamma	Wuxu Data Platform,20
FOLFIRI + Bevacizumab	5260.49	3945.37	6575.61	gamma	Wuxu Data Platform,20; CSCO guideline,16
Costs of administration ($/cycle)					
Best supportive care	734.58	550.94	918.23	gamma	22
Laboratory testing	21.66	16.25	27.08	gamma	National public hospital pricing standards in China
Imaging examination	45.835	34.38	57.29	gamma	National public hospital pricing standards in China
End of life care(one-time cost)	6633.22	4974.92	8291.53	gamma	22
Costs associated with grade ≤3 AEs ($/event)					
Neutropenia	1114.83	836.12	1393.54	gamma	23
Leukopenia	836.12	627.09	1045.15	gamma	24
Thrombocytopenia	975.47	731.60	1219.34	gamma	25
Diarrhea	10487	7865.25	13108.75	gamma	25
Infusion-related reaction	209.03	156.77	261.29	gamma	25
Dermatitis acneiform	139.35	104.51	174.19	gamma	25
Skin reaction	167.22	125.42	209.03	gamma	26
Electrolyte disturbance	348.38	261.29	435.48	gamma	25
Disutility associated with grade ≤3 AEs					
Disutility associated with neutropenia	0.15	0.11	0.19	beta	23
Disutility associated with leukopenia	0.12	0.09	0.15	beta	24
Disutility associated with thrombocytopenia	0.13	0.10	0.16	beta	24
Disutility associated with diarrhea	0.08	0.06	0.10	beta	25
Disutility associated with infusion-related reaction	0.05	0.04	0.06	beta	25
Disutility associated with dermatitis acneiform	0.04	0.03	0.05	beta	25
Disutility associated with skin reaction	0.06	0.05	0.08	beta	26
Disutility associated with electrolyte disturbance	0.1	0.08	0.13	beta	30
Utilities					
Utility of progression-free survival	0.78	0.59	0.98	beta	Supplementary **Table 3**
Utility of progressed disease	0.60	0.45	0.75	beta	
Discount rate	0.045	0.000	0.050	beta	17
Grade ≤3 adverse event incidence					
**Incidence of adverse events –**					
**A140 group**					
Neutropenia	0.37	0.28	0.46	beta	14
Leukopenia	0.14	0.11	0.18	beta	
Thrombocytopenia	0.03	0.02	0.04	beta	
Diarrhea	0.04	0.03	0.05	beta	
Infusion-related reaction	0.03	0.02	0.04	beta	
Dermatitis acneiform	0.04	0.03	0.05	beta	
Skin reaction	0.08	0.06	0.10	beta	
Electrolyte disturbance	0.07	0.05	0.09	beta	
Incidence of adverse events –					
Cetuximab group					
Neutropenia	0.44	0.33	0.55	beta	14
Leukopenia	0.19	0.14	0.24	beta	
Thrombocytopenia	0.04	0.03	0.05	beta	
Diarrhea	0.06	0.05	0.08	beta	
Infusion-related reaction	0.06	0.05	0.08	beta	
Dermatitis acneiform	0.03	0.02	0.04	beta	

Drug acquisition costs were based on median national bid-winning prices from January to June 2025 obtained from the Wuxu Data Platform. For cetuximab-based regimens, initial-cycle costs reflected the loading dose administered during the first 14-day cycle, whereas subsequent-cycle costs reflected maintenance dosing from the second cycle onward.

### 2.6 Sensitivity analysis

One-way sensitivity analysis and PSA were conducted to evaluate the robustness of the model results and assess the impact of parameter uncertainty on the incremental cost-effectiveness ratio (ICER).

In the one-way sensitivity analysis, parameter ranges were primarily based on reported 95% confidence intervals. For parameters without available variance estimates, a variation of ±25% from the base-case value was assumed. Results were presented using tornado diagrams to identify parameters with the greatest influence on the ICER.

In the PSA, 1,000 second-order Monte Carlo simulations were performed using the predefined parameter distributions. Cost parameters were assigned gamma distributions, whereas utility values and AE incidence probabilities were assigned beta distributions. Simulation outputs were used to generate cost-effectiveness planes and CEACs.

### 2.7 Economic evaluation of CMAB009 and exploratory contextual cross-trial assessment

To further evaluate the economic implications of EGFR-targeted biosimilar agents in China, two additional analyses were conducted.

First, model inputs for the CMAB009 analysis were derived from its published phase III trial NCT03206151, which compared CMAB009 plus FOLFIRI with FOLFIRI alone in Chinese patients with RAS/BRAF wild-type mCRC. Survival inputs, including PFS and OS, were derived from the published Kaplan–Meier curves and extrapolated using the same parametric survival modeling approach as described above. Grade ≥3 AEs with an incidence of ≥2% in at least one treatment group were included, and the corresponding incidence rates were applied to both treatment groups. These events included neutropenia, leukopenia, thrombocytopenia, diarrhea, stomatitis, hypokalemia, dermatitis, and anemia. Detailed drug costs, AE-related costs, utility parameters, AE incidence rates, parameter ranges, and distribution assumptions for this analysis are provided in **Supplementary Table 2**.

Second, an exploratory contextual cross-trial assessment was conducted to describe the potential economic implications of available cetuximab biosimilars in China. This assessment used evidence from two independent domestic phase III trials: NCT04835142 for A140 plus mFOLFOX6 and NCT03206151 for CMAB009 plus FOLFIRI. Given the absence of head-to-head evidence, the lack of a common comparator arm, and differences in chemotherapy backbones, this assessment was not designed or interpreted as a formal comparative effectiveness or cost-effectiveness analysis.

A formal network meta-analysis was not feasible because the two trials did not share a common comparator. Similarly, a matching-adjusted indirect comparison could not be performed because individual patient-level data and sufficient aggregate covariates for population reweighting were not available from the published reports. Therefore, the exploratory assessment was restricted to scenario analyses examining how alternative assumptions regarding survival outcomes and adverse event-related costs could influence modeled economic outcomes.

Scenario analyses were conducted as conservative stress tests rather than statistical adjustments for cross-trial heterogeneity. Survival outcomes for CMAB009 were reduced by 10% and 20%, AE-related costs for CMAB009 were increased by 50%, and a worst-case scenario combined a 20% survival reduction with a 50% increase in AE-related costs. Survival reduction scenarios were implemented by proportionally reducing CMAB009 survival probabilities at each model cycle while preserving the original parametric curve structure. The AE cost increase scenario was implemented by applying a multiplicative factor of 1.50 to all grade ≥3 AE-related management costs for CMAB009, while AE incidence rates and health-state utilities were kept unchanged. These analyses were intended to evaluate the sensitivity of modeled economic outcomes to unfavorable assumptions for CMAB009 and should not be interpreted as evidence of comparative clinical or economic superiority between CMAB009 and A140.

## 3 Results

### 3.1 Trial population characteristics and survival model fitting

In the NCT04835142 trial population, the median age was 59 years in the A140 group and 58 years in the reference cetuximab group. The mean body surface area and sex-weighted mean body weight were 1.7 m² and 63.7 kg, respectively. In the CMAB009 clinical trial population, the mean body surface area and sex-weighted mean body weight were 1.7 m² and 63.7 kg, respectively.

The reconstructed KM curves were visually consistent with the original trial curves (**Supplementary Figure 1**). For the primary analysis, the gamma distribution was selected for PFS in the reference cetuximab group, the Weibull distribution for PFS in the A140 group, and the Weibull distribution for OS in both groups. The fitted and extrapolated survival curves are shown in **Supplementary Figure 2**, and the AIC and BIC values are summarized in **Supplementary Table 1**.

### 3.2 Base-case analysis results

The base-case analysis (**Table 2**) showed that reference cetuximab plus chemotherapy was associated with higher total costs and slightly improved health outcomes compared with A140 plus chemotherapy. The incremental cost and incremental QALYs were USD 14,894.40 and 0.08 QALYs, respectively, resulting in an ICER of USD 193,033.41/QALY. At both predefined willingness-to-pay thresholds, reference cetuximab plus chemotherapy was not considered cost-effective compared with A140 plus chemotherapy.

**Table 2:** Base-case analysis results.

Group	Total cost	Total QALY	Incremental cost	Incremental QALY	ICER
TG	231,648.11	1.53	—	—	—
CG	246,542.51	1.60	14,894.40	0.08	193,033.41

TG, A140 plus chemotherapy group; CG, cetuximab plus chemotherapy group. Incremental costs, incremental QALYs, and ICERs were calculated for the CG strategy compared with the TG strategy.

### 3.3 Sensitivity analysis

**Figure 2:**
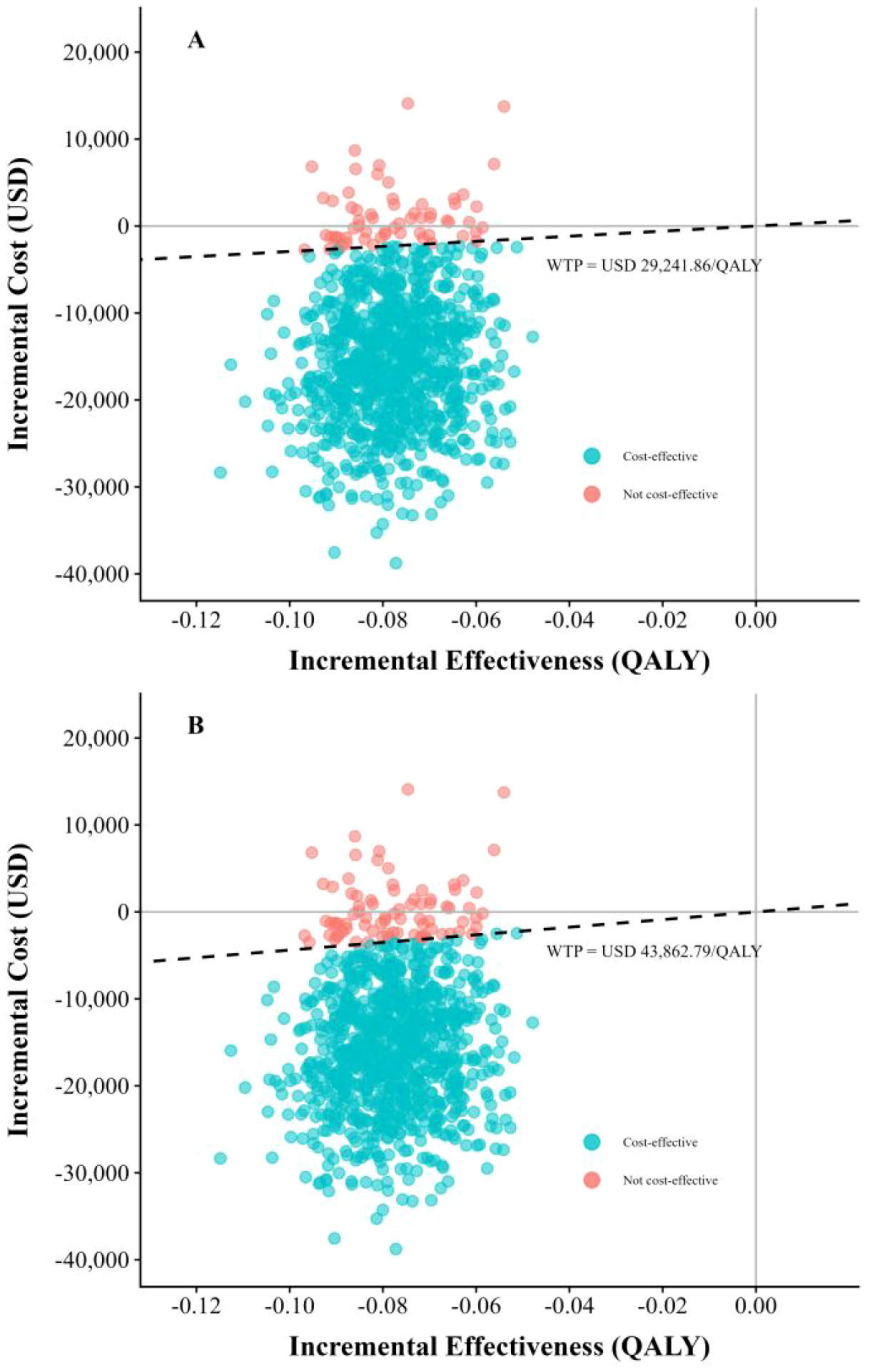
Cost-effectiveness plane from the probabilistic sensitivity analysis (PSA). Each dot represents one Monte Carlo simulation showing the joint distribution of incremental costs and incremental QALYs for A140 plus chemotherapy versus reference cetuximab plus chemotherapy. **(A)** shows the results at a WTP threshold of USD 29,241.86/QALY, and **(B)** shows the results at a WTP threshold of USD 43,862.79/QALY. The dashed line represents the corresponding WTP threshold. Teal dots indicate simulations in which A140 plus chemotherapy was cost-effective compared with reference cetuximab plus chemotherapy, whereas red dots indicate simulations in which it was not cost-effective.

**Figure 3:**
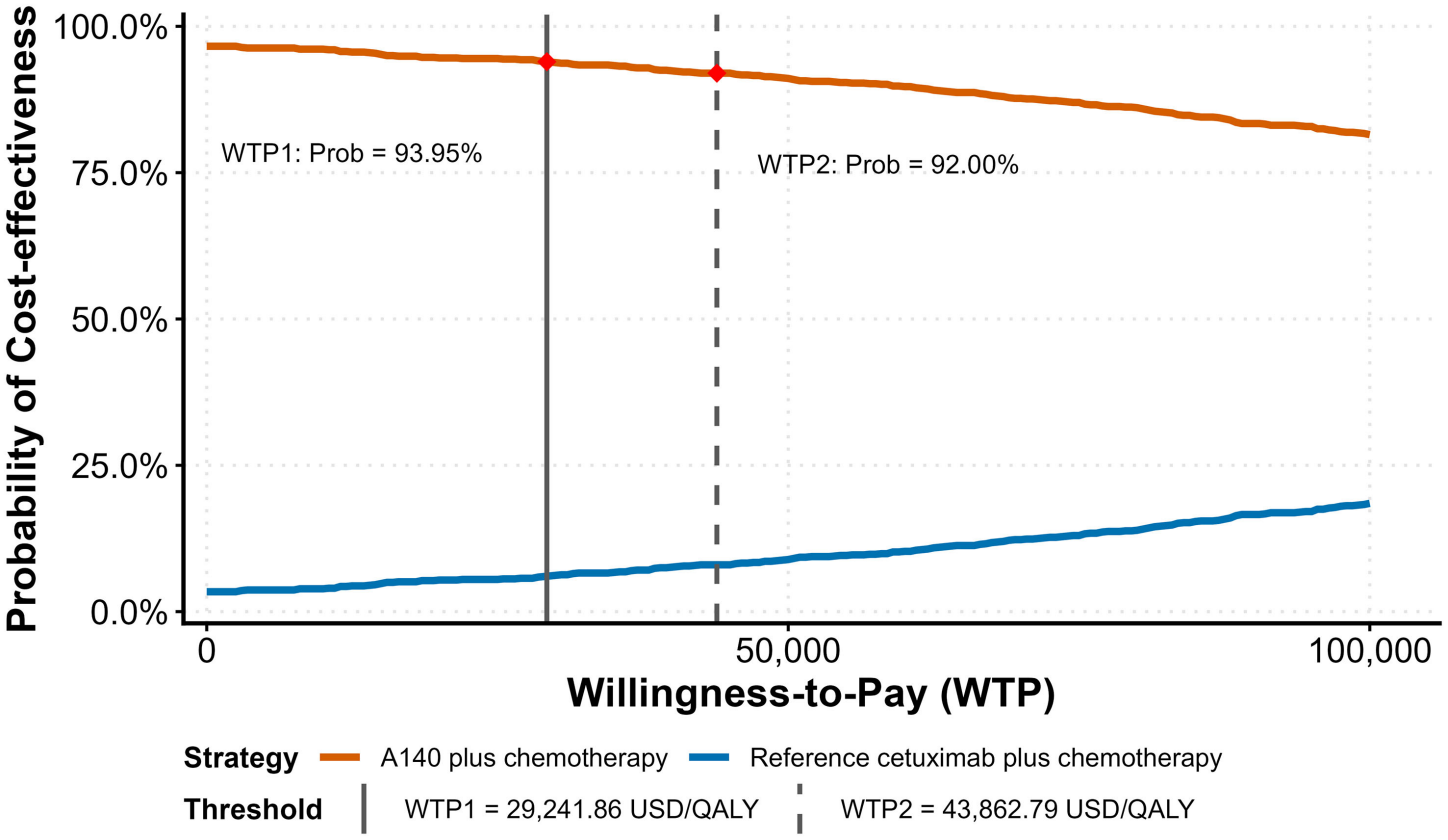
Cost-effectiveness acceptability curve (CEAC) for A140 plus chemotherapy and reference cetuximab plus chemotherapy. The curves show the probability of each strategy being cost-effective across different willingness-to-pay thresholds under probabilistic uncertainty. The vertical lines indicate the predefined WTP thresholds of USD 29,241.86/QALY and USD 43,862.79/QALY.

PSA results showed that most simulations were located in the southwest quadrant of the cost-effectiveness plane, indicating that A140 plus chemotherapy was generally associated with lower costs and slightly lower QALYs compared with reference cetuximab plus chemotherapy (**Figure 2**). At the predefined WTP thresholds of USD 29,241.86/QALY and USD 43,862.79/QALY, the CEAC showed that A140 plus chemotherapy had a higher probability of being cost-effective, with probabilities of 93.95% and 92.00%, respectively, whereas the corresponding probabilities for reference cetuximab plus chemotherapy were low (**Figure 3**).

**Figure 4:**
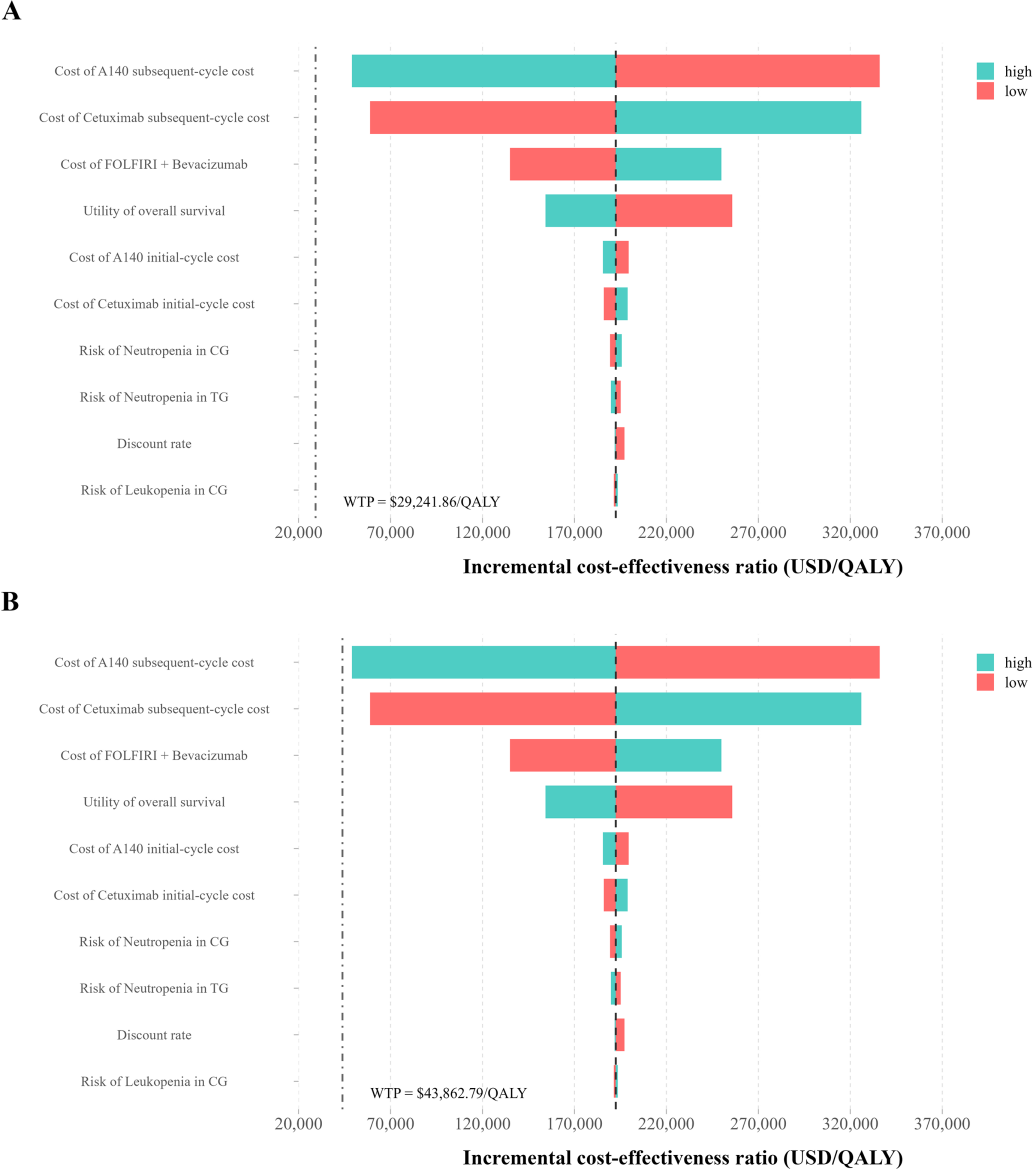
Tornado diagrams of one-way sensitivity analysis for the ICER. **(A, B)** represent WTP thresholds of USD 29,241.86/QALY and USD 43,862.79/QALY, respectively. The diagrams show the ten parameters with the greatest influence on the ICER, ranked by their impact on model results. The vertical dashed line indicates the corresponding WTP threshold.

The one-way sensitivity analysis showed that the ICER was most sensitive to the subsequent-cycle treatment costs of A140 and reference cetuximab, followed by the cost of second-line FOLFIRI plus bevacizumab and the utility parameter. In contrast, initial-cycle drug costs, discount rate, and grade ≥3 AE incidence parameters had relatively limited effects on the ICER. Across the tested parameter ranges, the ICER remained above both predefined WTP thresholds of USD 29,241.86/QALY and USD 43,862.79/QALY, indicating that reference cetuximab plus chemotherapy was unlikely to be cost-effective compared with A140 plus chemotherapy under one-way parameter uncertainty (**Figure 4**).

### 3.4 Economic evaluation and exploratory cross-trial analyses of EGFR-targeted biosimilars

#### 3.4.1 Cost-effectiveness analysis of CMAB009 plus FOLFIRI versus FOLFIRI alone

Based on a phase III clinical trial involving Chinese patients with RAS/BRAF wild-type metastatic colorectal cancer, CMAB009 plus FOLFIRI was associated with higher total costs than FOLFIRI alone (USD 214,917.27 vs. USD 148,659.96), while providing improved health outcomes (1.83 vs. 1.46 QALYs) (**Table 3**). The incremental cost and incremental QALYs were USD 66,257.31 and 0.37 QALYs, respectively, resulting in an ICER of USD 179,684.65/QALY. At the predefined WTP thresholds of USD 29,241.86/QALY and USD 43,862.79/QALY, CMAB009 plus FOLFIRI was not considered cost-effective compared with FOLFIRI alone.

**Table 3:** Cost-effectiveness results of CMAB009 plus FOLFIRI versus FOLFIRI alone.

Group	Total cost (USD)	Total QALY	Incremental cost (USD)	Incremental QALY	ICER/(USD/QALY)
CMAB009 Group	214917.27	1.83	66,257.3	0.37	179,684.65
Chemotherapy Group	148659.96	1.46			

#### 3.4.2 Exploratory contextual assessment of A140 and CMAB009

An exploratory contextual assessment was conducted to describe modeled economic outcomes for A140 and CMAB009, both in combination with standard chemotherapy, using evidence from two independent domestic phase III trials conducted in China. Because this assessment was based on unadjusted cross-trial evidence and no formal indirect treatment comparison was performed, the results should be interpreted as scenario-based economic findings rather than definitive comparative evidence.

In the exploratory base-case assessment, CMAB009 was associated with lower modeled total costs and marginally higher modeled QALYs than A140 (**Table 4**). Specifically, the total costs were USD 208,642.03 for CMAB009 and USD 229,435.30 for A140, while the corresponding QALYs were 1.56 and 1.53, respectively. When A140 was evaluated relative to CMAB009, the modeled incremental cost was USD 20,793.27 and the modeled incremental QALY was −0.02. However, because these estimates were derived from separate trials with different chemotherapy backbones and potential residual heterogeneity, this finding should not be interpreted as evidence of clinical or economic superiority.

**Table 4:** Exploratory cross-trial cost-effectiveness comparison between CMAB009 and A140.

Group	Total cost (USD)	Total QALYs	Incremental cost (USD)	Incremental QALYs	Economic outcome
A140 group	229,435.30	1.53	20,793.27	-0.02	Higher modeled cost and lower modeled QALYs
CMAB009 group	208,642.03	1.56	—	—	—

To explore structural uncertainty associated with the cross-trial assessment, scenario analyses were performed by varying survival outcomes and adverse event-related costs for CMAB009 (**Table 5**). When survival outcomes for CMAB009 were reduced by 10% and 20%, CMAB009 remained associated with lower modeled total costs but yielded slightly fewer QALYs than A140. Increasing AE-related costs for CMAB009 by 50% had only a limited impact on modeled economic outcomes, with CMAB009 remaining associated with lower costs and marginally higher QALYs. In the worst-case scenario, which combined a 20% reduction in survival outcomes with a 50% increase in AE-related costs for CMAB009, CMAB009 remained less costly but was associated with slightly fewer QALYs than A140.

**Table 5:** Exploratory scenario analyses of CMAB009 versus A140.

Scenario	Strategy	Total cost (USD)	Total QALYs	Incremental cost (USD)	Incremental QALYs	Economic outcome
Survival reduction (−10%)	A140	229435.30	1.53	22,502.82	0.04	Higher modeled cost and higher modeled QALYs
	CMAB009	206932.48	1.49	–	–	–
Survival reduction (−20%)	A140	229435.30	1.53	10,191.59	0.06	Higher modeled cost and higher modeled QALYs
	CMAB009	219243.71	1.47	–	–	–
Increased AE-related costs (+50%)	A140 group	229435.30	1.53	20,727.35	-0.03	Higher modeled cost and lower modeled QALYs
	CMAB009 group	208707.95	1.56	—	—	—
Worst-case scenario	A140 group	229435.30	1.53	10,132.25	0.06	Higher modeled cost and higher modeled QALYs
	CMAB009 group	219303.05	1.47	—	—	—

Overall, these scenario analyses suggested that modeled economic outcomes were more sensitive to assumptions regarding survival than to AE-related costs. These findings were intended to provide contextual economic information and should be interpreted cautiously because the analysis did not adjust for cross-trial differences in patient populations, censoring patterns, follow-up duration, or chemotherapy backbones.

Incremental costs and QALYs were calculated for A140 relative to CMAB009. These results were based on unadjusted cross-trial evidence and should not be interpreted as formal indirect comparative evidence.

Incremental costs and QALYs were calculated for A140 relative to CMAB009. These analyses were conducted as exploratory scenario-based assessments and should not be interpreted as formal indirect comparative evidence or as evidence of comparative clinical or economic superiority.

## 4 Discussion

This study evaluated the economic value of newly approved cetuximab biosimilars in China, including A140 and CMAB009, for first-line treatment of RAS/BRAF wild-type metastatic colorectal cancer (mCRC). The findings suggest that economic outcomes differed across biosimilar-based treatment strategies and were strongly influenced by drug pricing and assumptions regarding long-term survival outcomes. In the primary analysis based on the NCT04835142 head-to-head randomized trial, reference cetuximab plus chemotherapy was associated with modestly improved health outcomes compared with A140 plus chemotherapy, but at substantially higher costs. The resulting ICER exceeded the predefined willingness-to-pay (WTP) threshold, indicating that reference cetuximab was unlikely to be considered cost-effective under current pricing conditions in China. Sensitivity analyses showed that the model results were primarily driven by the acquisition costs of anti-EGFR agents and second-line treatment costs, whereas adverse event-related parameters had comparatively limited influence on the overall conclusions.

Accordingly, these analyses were intended to provide contextual information regarding the relative economic profiles of available biosimilar strategies rather than definitive comparative effectiveness evidence. Although the included phase III trials enrolled broadly similar patient populations, residual heterogeneity related to baseline characteristics, follow-up duration, and chemotherapy backbone could not be completely eliminated. To partially contextualize differences in chemotherapy backbones, the exploratory assessment was interpreted alongside current clinical guideline recommendations and published evidence suggesting broadly comparable efficacy between FOLFOX- and FOLFIRI-based regimens in first-line mCRC (6, 31, 32).

In the exploratory base-case comparison, CMAB009 was associated with lower total costs and marginally higher QALYs than A140. However, scenario analyses demonstrated that the economic conclusions were sensitive to assumptions regarding survival benefits. Under conservative survival reduction scenarios, CMAB009 remained less costly but became associated with slightly lower QALYs compared with A140. In contrast, increasing adverse event-related costs had only a limited impact on the economic outcomes. These findings suggest that uncertainty surrounding survival extrapolation and cross-trial comparability contributed more substantially to the results than uncertainty related to toxicity costs. From a pharmacoeconomic perspective, the findings highlight the importance of pricing in determining the economic value of anti-EGFR biosimilars in China. The WTP thresholds should be interpreted cautiously. In this study, two times China’s per capita GDP was used as the primary threshold, while three times the per capita GDP was considered as a more permissive scenario threshold. Although GDP-based thresholds are commonly used in economic evaluations, reliance on the upper threshold of three times GDP per capita has been criticized because it may not fully reflect affordability, budget impact, or health opportunity costs, particularly for high-cost biologic therapies (33, 34). Recent China-specific evidence also suggests that WTP per QALY thresholds vary by disease context and are generally close to approximately two times GDP per capita (35). In the primary head-to-head analysis, the ICER for reference cetuximab plus chemotherapy compared with A140 plus chemotherapy exceeded both the 2× and 3× GDP thresholds, supporting the robustness of the main conclusion under the predefined WTP thresholds.

In the context of China’s rapidly expanding biosimilar market and ongoing reforms in centralized procurement and reimbursement negotiations, acquisition prices are likely to remain a key determinant of the economic value of EGFR-targeted biosimilars (11, 36, 37). Therefore, China-specific procurement prices, reimbursement policies, and potential future price changes should be considered alongside modeled survival outcomes when assessing the value of cetuximab biosimilars in routine clinical practice. Although A140 demonstrated comparable efficacy to reference cetuximab in the clinical trial setting, its limited price advantage reduced its economic attractiveness under the current pricing structure. By comparison, the lower acquisition costs of CMAB009 contributed to its more favorable modeled economic profile in exploratory analyses, although these findings remain subject to uncertainty in cross-trial comparability and survival assumptions.

Several limitations should be acknowledged. Long-term survival outcomes were extrapolated beyond trial follow-up using parametric survival models, which may introduce structural uncertainty. The exploratory assessment involving A140 and CMAB009 was based on unadjusted cross-trial evidence and was not supported by formal indirect treatment comparison methods. A network meta-analysis was not feasible because the two trials did not share a common comparator arm. In addition, a matching-adjusted indirect comparison could not be performed because individual patient-level data and sufficient aggregate covariates required for population reweighting were not available from the published reports. Therefore, the exploratory scenario analyses cannot substitute for formal statistical adjustment and should not be interpreted as evidence of comparative clinical or economic superiority between CMAB009 and A140. Grade ≥3 AE-related costs and utility decrements were modeled as acute, one-time events in the first treatment cycle because the published clinical reports did not provide sufficient information on AE duration or long-term management costs. This assumption was applied consistently across treatment groups and was therefore not expected to introduce substantial systematic bias against biosimilar strategies, although residual uncertainty remains. Sensitivity analyses also showed that AE-related parameters had limited influence on the results. Utility values and some cost parameters were derived from published studies rather than directly collected from trial participants, which may affect external validity. Finally, the analysis was conducted from the perspective of the Chinese healthcare system and may not be generalizable to other healthcare settings.

## 5 Conclusion

Under current pricing conditions in China, reference cetuximab plus chemotherapy was unlikely to be cost-effective compared with A140 plus chemotherapy at both predefined WTP thresholds. Exploratory cross-trial analyses suggested that economic outcomes among available cetuximab biosimilars may differ because of variations in acquisition costs and assumptions regarding long-term survival. However, these findings should be interpreted cautiously because they were derived from indirect cross-trial comparisons rather than head-to-head evidence. Overall, the study provides model-based evidence regarding the economic value of newly approved cetuximab biosimilars in China and highlights the important role of pricing in determining their cost-effectiveness. Further comparative clinical and economic studies are warranted to better define the value of EGFR-targeted biosimilars in metastatic colorectal cancer.

## Data Availability

The model code and supplementary reproducibility materials are publicly available at: https://github.com/Huoliman/cetuximab-biosimilar-hta.
