# Supplementary material for: Economic evaluation of newly approved cetuximab biosimilars for first-line treatment of RAS/BRAF wild-type metastatic colorectal cancer in China
Source: Front Oncol. 2026 Jul 28;16:1901037. doi: 10.3389/fonc.2026.1901037 (PMC13459732; doi:10.3389/fonc.2026.1901037)
Supplement: Supplementary file 1 [file DataSheet1.docx]

**Economic evaluation of newly approved cetuximab biosimilars for first-line treatment of RAS/BRAF wild-type metastatic colorectal cancer in China**

Rui Feng^1†^, Liman Huo^2†^,Ping Liang^2^, Dong Li^2^, Rong Cui^2^, Ying Zheng^2*^and Qi Lv^*1^

1School of Disaster and Emergency Medicine, Tianjin University, Tianjin 300072, PR China；

2Department of Pharmacy, The Fourth Hospital of Hebei Medical University, Shijiazhuang050000, China；

^†^These authors have contributed equally to this work and share first authorship.

^*^CORRESPONDENCE

Qi Lv, Email：[lvqi@tju.edu.cn](mailto:lvqi@tju.edu.cn)；Ying Zheng，Email：hbykdxzy@hebmu.edu.cn

**Supplementary Material**

**A**

**B**

Figure S1. Digitized Kaplan–Meier curves for PFS and OS based on extracted data points. Kaplan–Meier curves for progression-free survival (PFS, Panel A) and overall survival (OS, Panel B) were reconstructed using data points digitized from published survival graphs. The A140 group received A140 plus mFOLFOX6, while the reference cetuximab group received reference cetuximab plus mFOLFOX6. These curves were reconstructed to visually assess the consistency between the extracted values and the original KM trends and to support subsequent survival extrapolation in the pharmacoeconomic model.

Table S1. Goodness-of-fit results for different parametric distributions of OS and PFS curves

| Parametric distribution | Fit criteria | OS curve | | PFS curve | |
| --- | --- | --- | --- | --- | --- |
|  |  | TG | CG | TG | CG |
| Exponential | AIC | 217.56 | 220.22 | 286.10 | 304.06 |
|  | BIC | 221.39 | 224.05 | 289.93 | 307.89 |
| Weibull | AIC | **190.18** | **198.48** | **221.97** | 236.41 |
|  | BIC | **197.85** | **206.14** | **229.64** | 244.07 |
| Gamma | AIC | 191.23 | 199.29 | 223.64 | **229.75** |
|  | BIC | 198.89 | 206.95 | 231.30 | **237.42** |
| Log-normal | AIC | 198.05 | 209.44 | 258.55 | 233.02 |
|  | BIC | 205.71 | 217.10 | 266.21 | 240.68 |
| Gompertz | AIC | 192.85 | 202.66 | 241.26 | 267.99 |
|  | BIC | 200.51 | 210.33 | 248.92 | 275.65 |
| Log-logistic | AIC | 192.57 | 199.32 | 221.94 | 225.14 |
|  | BIC | 200.24 | 206.98 | 229.61 | 232.80 |
| Generalized Gamma | AIC | 191.70 | 200.49 | 223.64 | 230.76 |
|  | BIC | 203.20 | 211.98 | 235.13 | 242.26 |
| FP1 | AIC | 189.77 | 197.70 | 221.61 | 229.67 |
|  | BIC | 197.43 | 205.37 | 229.27 | 237.33 |
| FP2 | AIC | 188.21 | 196.99 | 205.71 | 227.62 |
|  | BIC | 199.71 | 208.48 | 217.21 | 239.11 |
| RCS | AIC | 193.28 | 197.11 | 212.85 | 228.72 |
|  | BIC | 204.77 | 208.60 | 228.17 | 244.04 |
| RP-hazard | AIC | 190.18 | 198.48 | 212.30 | 225.94 |
|  | BIC | 197.85 | 206.14 | 227.63 | 241.27 |
| RP-odds | AIC | 192.26 | 199.01 | 211.74 | 225.14 |
|  | BIC | 215.25 | 210.51 | 227.07 | 232.80 |
| RP-normal | AIC | 191.76 | 200.41 | 211.79 | 226.67 |
|  | BIC | 210.91 | 219.57 | 227.12 | 242.00 |
| GAM | AIC | 192.24 | 196.95 | 212.63 | 228.35 |
|  | BIC | 199.90 | 207.84 | 227.10 | 243.96 |
| MCM | AIC | 546.12 | 545.36 | 352.27 | 345.68 |
|  | BIC | 557.62 | 560.68 | 371.43 | 364.84 |

Note: Bold and red-highlighted values indicate the parametric models selected for the base-case analysis after considering AIC, BIC, visual goodness-of-fit, and clinical plausibility of long-term extrapolation.

A

B

C

D

Figure S2. Fitted and extrapolated survival curves for PFS and OS based on parametric models. Panels A–D compare Kaplan–Meier (KM) survival curves with fitted and extrapolated curves generated using the selected parametric models based on AIC, BIC, visual goodness-of-fit, and clinical plausibility. Dashed black lines represent KM curves reconstructed from digitized trial data, while solid red lines indicate extrapolated survival curves based on parametric distributions extended to 120 months.

Panel A: PFS in the reference cetuximab group, modeled with a gamma distribution. Panel B: PFS in the A140 group, modeled with a Weibull distribution. Panel C: OS in the reference cetuximab group, modeled with a Weibull distribution. Panel D: OS in the A140 group, modeled with a Weibull distribution.

Table S2. Base-case parameters and distributions used in the Markov model for CMAB009 plus FOLFIRI versus FOLFIRI alone

| **Variable** | **Baseline value** | **Low** | **High** | **Distribution** | **Source** |
| --- | --- | --- | --- | --- | --- |
| **Costs of drugs (USD/cycle)** |  |  |  |  |  |
| CMAB009 initial-cycle cost | 1215.95 | 911.96 | 1519.93 | gamma | Wuxu Data Platform, 1 |
| CMAB009 subsequent-cycle cost | 929.87 | 697.41 | 1162.34 | gamma | Wuxu Data Platform, 1 |
| FOLFIRI | 596.94 | 447.71 | 746.18 | gamma | Wuxu Data Platform, 1 |
| m-FOLFOX6 + Bevacizumab | 3998.40 | 2998.80 | 4998.00 | gamma | Wuxu Data Platform, 1,2 |
| **Costs of administration (USD/cycle)** |  |  |  |  |  |
| Best supportive care | 734.58 | 550.94 | 918.23 | gamma | 3 |
| Laboratory testing | 22.30 | 16.72 | 27.87 | gamma | National public hospital pricing standards in China |
| Imaging examination | 62.71 | 47.03 | 78.39 | gamma | National public hospital pricing standards in China |
| End of life care (one-time cost) | 6633.22 | 4974.92 | 8291.53 | gamma | 3 |
| **Costs associated with grade ≥3 AEs (USD/event)** |  |  |  |  |  |
| Neutropenia | 1114.83 | 836.12 | 1393.53 | gamma | 4 |
| Leukopenia | 836.12 | 627.09 | 1045.15 | gamma | 5 |
| Thrombocytopenia | 975.47 | 731.61 | 1219.34 | gamma | 6 |
| Diarrhea | 278.71 | 209.03 | 348.38 | gamma | 7 |
| Stomatitis | 350.63 | 262.97 | 438.29 | gamma | 7 |
| Hypokalemia | 115.85 | 86.89 | 144.81 | gamma | 7 |
| Dermatitis (Rash) | 40.97 | 30.73 | 51.21 | gamma | 7 |
| Anemia | 691.87 | 518.90 | 864.83 | gamma | 7 |
| **Disutility associated with grade ≥3 AEs** |  |  |  |  |  |
| Disutility associated with stomatitis | 0.15 | 0.11 | 0.19 | beta | 7 |
| Disutility associated with hypokalemia | 0.05 | 0.04 | 0.06 | beta | 7 |
| Disutility associated with dermatitis (rash) | 0.10 | 0.08 | 0.13 | beta | 7 |
| Disutility associated with anemia | 0.09 | 0.06 | 0.11 | beta | 7 |
| Disutility associated with neutropenia | 0.15 | 0.11 | 0.19 | beta | 4 |
| Disutility associated with leukopenia | 0.12 | 0.09 | 0.15 | beta | 5 |
| Disutility associated with thrombocytopenia | 0.13 | 0.10 | 0.16 | beta | 6 |
| Disutility associated with diarrhea | 0.08 | 0.06 | 0.10 | beta | 7 |
| **Utilities** |  |  |  |  |  |
| Utility of progression-free survival | 0.78 | 0.59 | 0.98 | beta |  |
| Utility of progressed disease | 0.60 | 0.45 | 0.75 | beta |  |
| Discount rate | 0.045 | 0.000 | 0.050 | beta | 21 |
| **Grade ≥3 adverse event incidence** |  |  |  |  |  |
| **Incidence of adverse events – CMAB009 group** |  |  |  |  |  |
| Neutropenia | 0.57 | 0.43 | 0.71 | beta | 23 |
| Leukopenia | 0.33 | 0.25 | 0.41 | beta | 23 |
| Thrombocytopenia | 0.02 | 0.02 | 0.03 | beta | 23 |
| Diarrhea | 0.10 | 0.08 | 0.13 | beta | 23 |
| Stomatitis | 0.03 | 0.02 | 0.04 | beta | 23 |
| Hypokalemia | 0.04 | 0.03 | 0.05 | beta | 23 |
| Dermatitis | 0.06 | 0.05 | 0.08 | beta | 23 |
| Anemia | 0.05 | 0.04 | 0.06 | beta | 23 |
| **Incidence of adverse events – FOLFIRI group** |  |  |  |  |  |
| Neutropenia | 0.35 | 0.26 | 0.44 | beta | 23 |
| Leukopenia | 0.16 | 0.12 | 0.20 | beta | 23 |
| Thrombocytopenia | 0.01 | 0.01 | 0.01 | beta | 23 |
| Diarrhea | 0.12 | 0.09 | 0.15 | beta | 23 |
| Stomatitis | 0.01 | 0.01 | 0.01 | beta | 23 |
| Hypokalemia | 0.02 | 0.02 | 0.03 | beta | 23 |
| Dermatitis | 0.00 | 0.00 | 0.01 | beta | 23 |
| Anemia | 0.07 | 0.05 | 0.09 | beta | 23 |

Notes: Ranges for adverse event incidence probabilities were estimated using ±25% variation around the base-case values when corresponding confidence intervals were unavailable. The relatively high cost assigned to grade ≥3 diarrhea reflected hospitalization-related management and supportive care requirements reported in published oncology economic evaluations. The mFOLFOX6 plus bevacizumab cost was used to represent post-progression subsequent-line treatment in the CMAB009 model.

Table S3. Published utility estimates for progression-free survival and progressed disease in metastatic colorectal cancer used for utility synthesis

| **Clinical setting** | **PFS utility** | **PD utility** | **Notes** | **Source** |
| --- | --- | --- | --- | --- |
| MSI-H/dMMR metastatic colorectal cancer | 0.78 | 0.60 | Literature-derived utility inputs | 4,10 |
| Second-line metastatic colorectal cancer | 0.80 | 0.62 | Utility values reported in economic evaluations | 11,12 |
| Anti-EGFR-based first-line treatment | 0.79 | 0.61 | Based on previous cetuximab economic evaluations | 13 |
| FOLFOXIRI/FOLFOX-based treatment | 0.76–0.80 | 0.58–0.65 | Range used in scenario and sensitivity analyses | 14 |
| Third-line metastatic colorectal cancer | 0.75 | 0.58 | Utility values from later-line treatment studies | 5,8,15 |
| Bevacizumab-based treatment strategies | 0.78 | 0.60 | Derived from published economic evaluations | 16,17,18 |
| Chinese economic evaluations of cetuximab biosimilars | 0.85 | 0.68 | Higher utilities reported in Chinese populations | 19 |
| Cellular immunotherapy plus chemotherapy | 0.85 | 0.65 | Utility estimates from combination treatment studies | 20 |
| Refractory metastatic colorectal cancer | 0.76 | 0.59 | Chinese population-based utility estimates | 3 |

**Data and Code Availability**

The model parameter tables, transition probability matrices, and R scripts used for the present pharmacoeconomic analysis are publicly available at: <https://github.com/Huoliman/cetuximab-biosimilar-hta>

These materials are provided to improve methodological transparency and reproducibility.

**References**

1 Wuxu Data Technology Co., Ltd. (2025). National bid-winning drug price data (January–June 2025) [EB/OL]. Shanghai: Wuxu Data Platform. [cited 2025 Jul 21]. Available from: https://www.wuxuwang.com/home?html=0

2 Mi M, Weng S, Xu Z, Hu H, Wang Y, Yuan Y. CSCO guidelines for colorectal cancer version 2023: Updates and insights. Chin J Cancer Res. 2023;35(3):233–238. doi:10.21147/j.issn.1000‑9604.2023.03.021.

3.Li Z, Pan Z, Zhang L, He R, Jiang S, Xu C, Lu F, Zhang P, Li B. End‑of‑life cost and its determinants for cancer patients in urban China: a population‑based retrospective study. BMJ Open. 2019;9(3):e026309. doi:10.1136/bmjopen-2018-026309

4 Liu T, Liu S, Guan S, Tai Y, Jin Y, Dong M. Cost-effectiveness analysis of pembrolizumab versus chemotherapy for microsatellite instability-high or mismatch repair-deficient metastatic colorectal cancer. J Chemother. 2023 Dec;35(8):745-752. doi: 10.1080/1120009X.2022.2162220.

5 Zhong J, Liu Y, Fu Q, Huang D, Gong W, Zou J. Cost-Effectiveness Analysis of Regorafenib versus Other Third-Line Treatments for Metastatic Colorectal Cancer. Cancer Manag Res. 2024 Jun 5;16:593-602. doi: 10.2147/CMAR.S464831.

6 Liu T, Yao R, Tong Z, Dong M, Zhong L. Cost-effectiveness analysis of capecitabine versus active monitoring in stable or responding metastatic colorectal cancer after 16 weeks of first-line therapy. Ann Med. 2025 Dec;57(1):2529573. doi: 10.1080/07853890.2025.2529573.

7 Huang Z, Zhou L, Zheng H, Zhan M. Cost-effectiveness analysis of fruquintinib in Chinese patients with refractory metastatic colorectal cancer. Int J Clin Pharm. 2024 Aug;46(4):872-880. doi: 10.1007/s11096-024-01721-1.

8 Shi HY, Chen YC, Huang CW, Li CC, Su WC, Chang TK, Chen PJ, Yin TC, Tsai HL, Wang JY. Effectiveness and Cost-Utility Analysis of Different Doses of Irinotecan Plus Bevacizumab in Patients With Metastatic Colorectal Cancer: A Long-Term and Prospective Cohort Study. Front Oncol. 2022 Mar 14;12:756078. doi: 10.3389/fonc.2022.756078.

9 Nafees B, Stafford M, Gavriel S, Bhalla S, Watkins J. Health state utilities for non small cell lung cancer. Health Qual Life Outcomes. 2008 Oct 21;6:84. doi: 10.1186/1477-7525-6-84.

10Pembrolizumab for untreated metastatic colorectal cancer with high microsatellite instability or mismatch repair deficiency. London: National Institute for Health and Care Excellence (NICE); 2021 Jun 23.

11Hoyle M, Crathorne L, Peters J, Jones-Hughes T, Cooper C, Napier M, et al. The clinical effectiveness and cost-effectiveness of cetuximab (mono- or combination chemotherapy), bevacizumab (combination with non-oxaliplatin chemotherapy) and panitumumab (monotherapy) for the treatment of metastatic colorectal cancer after first-line chemotherapy: a systematic review and economic model. Health Technol Assess. 2013;17(14):1–237. doi:10.3310/hta17140.

12 National Institute for Health and Care Excellence (NICE). Aflibercept in combination with irinotecan and fluorouracil-based therapy for treating metastatic colorectal cancer that has progressed following prior oxaliplatin-based chemotherapy. Technology appraisal guidance [TA307]. London: NICE; 2014.

13Wang H, Huang L, Gao P, Zhu Z, Ye W, Ding H, Fang L. Cost-effectiveness analysis of cetuximab combined with chemotherapy as a first-line treatment for patients with RAS wild-type metastatic colorectal cancer based on the TAILOR trial. BMJ Open. 2020 Feb 12;10(2):e030738. doi: 10.1136/bmjopen-2019-030738.

14 Cremolini C, Loupakis F, Antoniotti C, Lupi C, Sensi E, Lonardi S, Mezi S, Tomasello G, Ronzoni M, Zaniboni A, Tonini G, Carlomagno C, Allegrini G, Chiara S, D'Amico M, Granetto C, Cazzaniga M, Boni L, Fontanini G, Falcone A. FOLFOXIRI plus bevacizumab versus FOLFIRI plus bevacizumab as first-line treatment of patients with metastatic colorectal cancer: updated overall survival and molecular subgroup analyses of the open-label, phase 3 TRIBE study. Lancet Oncol. 2015 Oct;16(13):1306-15. doi: 10.1016/S1470-2045(15)00122-9.

15 Huang LZ, Chen YQ, Gu HY, Chen Y. Cost-effectiveness analysis of trifluridine/tipiracil combined with bevacizumab vs. monotherapy for third-line treatment of colorectal cancer. Front Public Health. 2024 Nov 13;12:1465898. doi: 10.3389/fpubh.2024.1465898.

16 National Institute for Health and Care Excellence (NICE). Regorafenib for previously treated metastatic colorectal cancer. Technology appraisal guidance [TA866]. London: NICE; 2023.

17 Goldstein DA, Ahmad BB, Chen Q, Ayer T, Howard DH, Lipscomb J, El-Rayes BF, Flowers CR. Cost-Effectiveness Analysis of Regorafenib for Metastatic Colorectal Cancer. J Clin Oncol. 2015 Nov 10;33(32):3727-32. doi: 10.1200/JCO.2015.61.9569. Epub 2015 Aug 24.

18 National Institute for Health and Care Excellence (NICE). Bevacizumab in combination with oxaliplatin and either fluorouracil plus folinic acid or capecitabine for the treatment of metastatic colorectal cancer. Technology appraisal guidance [TA212]. London: NICE; 2010.

19 Tong R, Yang M, Zhang W, Zhou M, Wang L, Lu Y, Chang F. Network meta-analysis and cost-effectiveness analysis comparing Cetuximab-β and Cetuximab for Chinese patients with RAS/BRAF wild-type metastatic colorectal cancer. Front Pharmacol. 2025 Sep 15;16:1568385. doi: 10.3389/fphar.2025.1568385.

20 Huo L, Liang P, Duan Y, Xu Y, Tang J, Lv Q, Feng R. Adoptive cellular immunotherapy combined with chemotherapy versus chemotherapy alone in Chinese patients with metastatic colorectal cancer: a cost-effectiveness analysis to inform drug pricing. Front Oncol. 2025 Jun 9;15:1590319. doi: 10.3389/fonc.2025.1590319.

21 Liu, G. Guidelines for Pharmacoeconomic Evaluations in China (2020 edition). Beijing: China Market Press. pp. 3–5. (in Chinese)

22 Liu R, Yin X, Bai Y, Zhong H, Ye F, Wang Q, etal. A140 plus mFOLFOX6 compared with cetuximab plus mFOLFOX6 for first‑line RAS wild‑type metastatic colorectal cancer: a randomized clinical trial. Eur J Cancer. 2025;226:115566. doi:10.1016/j.ejca.2025.115566

23 Shi Y, Ba Y, Wang J, Xiong J, Gu K, Chen Y, et al. First-line treatment of anti‑EGFR monoclonal antibody cetuximab β plus FOLFIRI versus FOLFIRI alone in Chinese patients with RAS/BRAF wild‑type metastatic colorectal cancer: a randomized, phase 3 trial. Signal Transduct Target Ther. 2025;10(1):147. doi:10.1038/s41392-025-02229-4
